# A Novel Graphite-Based Sorbent for Oil Spill Cleanup

**DOI:** 10.3390/ma15020609

**Published:** 2022-01-14

**Authors:** Marco Vocciante, Alessandra De Folly D’Auris, Andrea Pietro Reverberi

**Affiliations:** 1 Department of Chemistry and Industrial Chemistry, University of Genova, Via Dodecaneso 31, 16146 Genova, Italy; reverb@dichep.unige.it; 2 Eni S.p.A., Environmental & Biological Laboratories, Via Maritano 26, 20097 San Donato Milanese, Italy; alessandra.de.folly.dauris@eni.com

**Keywords:** water remediation, hydrocarbons removal, selective adsorption, innovative adsorbents, super-expanded graphite, sustainable remediation

## Abstract

The performance of an innovative material based on expanded graphite, Grafysorber^®^ G+ (Directa Plus), has been tested through laboratory, tank, and confinement tests for oil removal in case of an oil spill and water treatment. In addition to the ability to retain oil, the possibility of reusing this material after regeneration via squeezing was also evaluated. As a comparison, the same experimental tests were conducted using polypropylene flakes (PP), the material currently most used to deal with spill accidents. Oils with different chemical and physical properties were used, namely kerosene, diesel, and crude oil. From the laboratory tests, the capacity of Grafysorber^®^ G+ to retain oil was found to be directly proportional to the viscosity of the latter, with adsorption values ranging from 76.8 g/g for diesel to 50.8 g/g for kerosene, confirming the potential of the innovative material compared to the PP. Cyclical use tests have confirmed certain reusability of the material, even if its adsorbent capacity decreases significantly after the first cycle and continues to decrease in subsequent cycles, but a less marked manner. Finally, some considerations based on the adsorption capacities were found to suggest that the adoption of the new material is also economically preferable, resulting in savings of 20 to 40% per kg of hydrocarbon treated.

## 1. Introduction

The term oil spill refers to any accidental or intentional release of liquid hydrocarbons into the environment. These events can vary greatly in type and entity, but they are generally rather difficult to stem, and often responsible for catastrophic effects on the environment.

Large spills have a huge environmental impact, strongly affecting in the long term the biological resources concerned [1], but minor spills are not to be underestimated, since they can still lead to severe consequences due to the displacement of contaminants in the soil [2,3] and from this to other matrices [4].

The growing attention of society to environmental issues, together with the stringent policies put in place by governments, are helping to reduce the frequency of significant spills over time [5]; nevertheless, events of devastating magnitude still take place, such as the recent Orange County oil spill in 2021 [6], the MV Wakashio (Mauritius) oil spill in 2020 [7] or the MT Dawn Kanchipuram accident in 2017 [8], to name a few.

To address oil spills and related problems, numerous approaches have been developed exploiting different principles of removal [9]. Among the physical interventions, sorbents, in particular, are classified as hybrid systems, as they can be used as (passive) containment or for the (active) removal of contaminants and can be particularly effective in recovering oil traces from the environment [10].

Indeed, adsorption is well known as a simple and effective approach to deal with aqueous solutions affected by various types of contamination, both organic [11] and inorganic [12,13], possibly by exploiting low-cost adsorbent materials from waste [14], with a dual benefit for the environment, according to the European dictates of the Circular Economy and the “near-zero discharge” of hazardous waste (see, for example [15,16,17]). By virtue of this, absorbent materials have recently attracted considerable attention in managing oil spills, combining the simple use with other advantages [18], including the possibility of recovering the adsorbed oil.

Nowadays, materials of all kinds are exploited to produce oil sorbents—from organics such as bark, straw, wool, or even dog fur and human hair [19], to inorganic such as alumina [20], and synthetic compounds such as polypropylene (PP) and other polymers—as long as several requirements are met, including high absorption and retention capacities, good selectivity, low cost, and biodegradability, to name a few [21].

Natural sorbents generally suffer from lower efficiency and worse mechanical and practical use characteristics compared to synthetic ones, but they balance these aspects with cost-effectiveness and low environmental impact (biodegradability) [22]. These are generally found as loose materials that can be used as it is, or organized according to need in cushions, booms, absorbent sheets, etc.

As mentioned, synthetic materials generally have better performance; among these, PP is the most widely used to date and is often taken as a reference standard. However, as an adsorbent material, PP presents several critical issues (lack of biodegradability, difficult recovery of the adsorbed oil, expensive landfill disposal of the exhausted material [23]), as well as application limitations, resulting in inefficient in adsorbing hydrocarbons present at low concentrations or as an iridescent monolayer.

Considering all of the above, the development of natural-based oil absorbents which, while preserving their pros (for example biodegradability), have adsorption performance comparable to those of the best synthetic materials, would be of great interest, as extremely relevant for improving the sustainability of the intervention and in line with numerous other solutions belonging to “green remediation”, such as the replacement of toxic reagents with low impact ones [24] and the abatement of pollutants with photocatalytic processes [25,26].

In this context, a promising option is represented by expanded graphite (EG).

The effectiveness of EG for “oil on water” or “pure oil” sorption was first discovered by the research group of Inagaki and Toyoda, which deeply investigated several aspects such as the maximum adsorption capacity for different oils [27] or the possible oil recovery and EG reuse [28]. Its buoyancy [29] and adsorption performance poorly affected by salinity [30] represent further undeniable advantages, confirming its use under seawater conditions [31], without the need to add additives or reaction aids [32,33]. Furthermore, the EG adsorption capacity increases with the expansion rate of the material [34], thus suggesting a strategy to further improve performances.

Starting from this observation, an innovative oleophilic/hydrophobic material with high adsorption capacity was recently developed, named Grafysorber^®^ G+ (Directa Plus, Como, Italy). The proprietary technology G+ confers unique characteristics to the material compared to the conventional EG, resulting in a peculiar three-dimensional structure—in which layers of graphene are weakly bound assuming an accordion-like shape—particularly effective towards the oil phase [9]. Completely free of additives, Grafysorber^®^ G+ can be used in loose form (e.g., for industrial wastewater treatments) as it can be easily handled and removed once saturated. Instead, it is intended for use in confined shapes (cushions, booms) for oil spill situations.

The peculiar characteristics of Grafysorber^®^ G+ make it a completely innovative and very promising solution compared to other EG-based adsorbents. Moreover, avoiding any chemical treatments with organic solvents or acids, but just on physics, the production process of G+ is also sustainable and safe for the environment.

However, given the novelty of this material, there are still few available experiments on its characteristics and its adsorption efficacy in the presence of different hydrocarbon compounds. In a recent work [9] the performances of the new material within adsorbent devices (booms and pillows) have been directly tested in the field for reasons of expediency, to possibly improve the remediation activity of a site of interest polluted by a strong presence of hydrocarbons, where PP sorbents were already adopted. This allowed for a direct comparison between the real in situ performances of the two materials, but the information obtained has little transferability due to the complex and site-specific contamination.

In this contribution, further laboratory investigations were carried out to deepen basic knowledge of this new material to improve general efficiency in the field. Three types of tests were performed. Initially, an evaluation of performances was carried out in the laboratory using small amounts of material and oil, in static conditions. Then, tests were conducted on a larger scale using a 1 × 5 m tank with wave application. Finally, containment tests were performed in the laboratory.

## 2. Materials and Methods

### 2.1. Adsorbent Materials

Grafysorber^®^ G+ is innovative expanded graphite with an apparent density between 2.3 and 3.5 g/L, a technological product patented by Directa Plus S.p.A. (Lomazzo (CO), Italy) [35].

**Figure 1:**
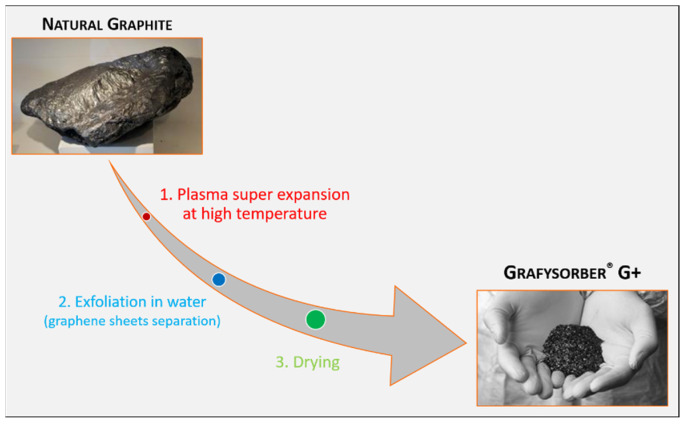
The production process of Grafysorber^®^ G+.

G+ is made up of Pristine Graphene Nanoplatelets (GNPs) by using a unique top-down approach that does not involve the use of chemicals nor solvents and is only based on physics. Starting from natural graphite, the production process takes place in three steps: (i) a plasma super expansion at high temperature (around 10,000 °C), which results in a super-expanded graphite; (ii) the exfoliation of this super-expanded graphite in water, where graphene sheets are separated; (iii) a drying step, where a super fine powder made from GNPs is obtained (Figure 1). In this way, every gram of graphite is transformed into a gram of G+, pushing the graphite expansion ratio to its maximum, quantifiable in about 300 units against average values 100 c.a. obtainable through standard methods [36,37].

In addition, by avoiding any chemical treatments with organic solvents or acids, and just exploiting water, temperature, and pressure to reduce the graphite thickness to the nanometric level, the production process is sustainable and safe for the environment. By consequence, G+ is completely free of additives (chemicals or nanometals used in other expanded graphite production processes) that could remain in the final graphene-enhanced product, thus potentially representing a health hazard.

**Figure 2:**
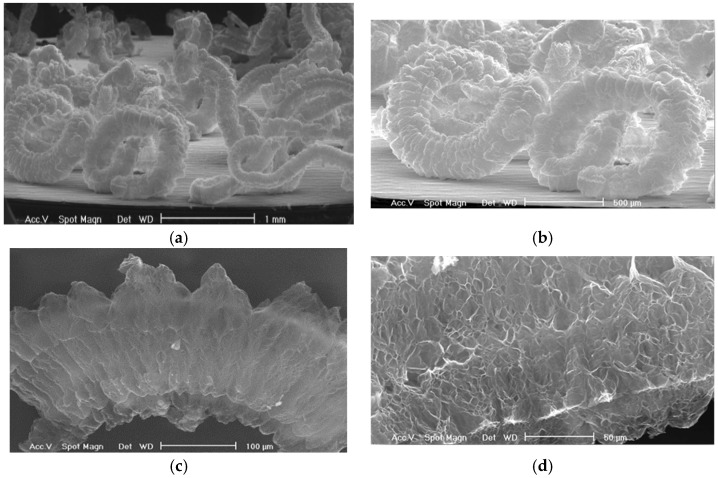
Characterization of the Grafysorber by Scanning Electron Microscopy (SEM) analysis at different magnifications (**a**) 1 mm; (**b**) 500 µm; (**c**) 100 µm; (**d**) 50 µm.

The expanded form obtained is a structure similar to an accordion (60–300 µm in diameter and length up to 5–6 mm), with few layers of graphite weakly bound along the edges (Figure 2), resulting in a low apparent density, which translates into a considerable adsorption capacity of the loose material [38].

This structure leads to another important innovation compared to conventional expanded graphite. In fact, in many cases, graphene particles are obtained, which are nanometric in all three dimensions. Conversely, G+ is made of graphene nanoplatelets with a lateral dimension in the micrometer range. Only the thickness is nanometric. This is a key feature to properly categorize graphene morphology, which results still in the range of 3D and does not present all the toxicological issues correlated with a nanomaterial, as confirmed by numerous certifications issued by independent laboratories [39].

Therefore, Grafysorber^®^ G+ is chemically and biologically inert; in addition, the highly crystalline nature guarantees the thermal stability of the material up to 600 °C in the air (1000 °C under an inert atmosphere), and provides a highly hydrophobic behavior.

The adsorption phenomenon takes place thanks to Van der Waals forces between oil molecules and the rough surface of each particle, where the spaces between each particle give an important contribution to the entrapment of more viscous oils. Because of the physical nature of the adsorption, the trapped oil can be easily recovered from the material by mechanical pressing.

Visually, Grafysorber^®^ G+ appears as a coarse powder, and the extreme lightness conferred by its three-dimensional, accordion-like, quaternary structure makes it difficult to use as it is for oil spills in open environments (e.g., sea, lakes, and rivers). In such cases, the material is confined to a non-woven fabric (NWF) that allows the oil to penetrate inside it. Specific assessment on properties of the NWF used (made of polypropylene) did not show any evidence of oil retention by the NWF.

Considering this, small cushions of 10 × 10 cm size containing about 0.9 g of material for laboratory tests were packaged. Similarly, mini booms of 50 cm in length and 7 cm in diameter, containing about 23 g of Grafysorber^®^ G+, and cushions of 50 × 50 cm in size containing 65 g of Grafysorber^®^ G+ were prepared for containment and tank tests, respectively.

By way of comparison, the same pillow and boom structures containing polypropylene (and a small percentage of polystyrene to increase its buoyancy) were prepared, being PP in flakes one of the materials currently used to stem spills in surface waters.

### 2.2. Laboratory Tests

To evaluate a possible relationship between oil density and absorption capacity and to have a picture as complete as possible regarding the performance of the material, three different oils were tested: diesel oil, kerosene, and crude oil, whose properties are reported in Table 1.

Crude oil and diesel come from a refinery, while kerosene was purchased from Sigma Aldrich.

**Figure 3:**
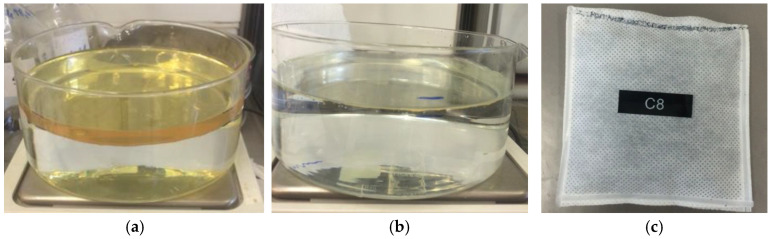
(**a**) Initial condition of test 2; (**b**) Initial condition of test 3; (**c**) Pillow used for laboratory tests.

For each oil, tests were performed under different operating conditions:

1. Water only;
2. Water + oil (oil thickness of 1 cm on 5 cm of water, Figure 3a);
3. Water + oil (surface iridescence, Figure 3b).

The laboratory equipment used for the tests is comprised of a crystallizer, a sieve with a 1.7 mm mesh used to evaluate the loss of absorbed product, and an analytical balance.

For these tests, pillows with a size of 10 × 10 cm and containing about 0.9 g of Grafysorber^®^ G+ were used, such as the one shown in Figure 3c. All tests were repeated using similar pillows but containing alternately G+ and PP flakes for comparison.

To assess the possible re-use of the material, pillows are squeezed manually after saturation of the same and then placed back in contact with the oil. This option, made possible by the purely physical adsorption underlying the functioning of the G+, allows for easy oil recovery from the saturated G+ directly in the field, without the aid of complex equipment or materials, and therefore represents the most practical, fast, and economical solution.

**Figure 4:**
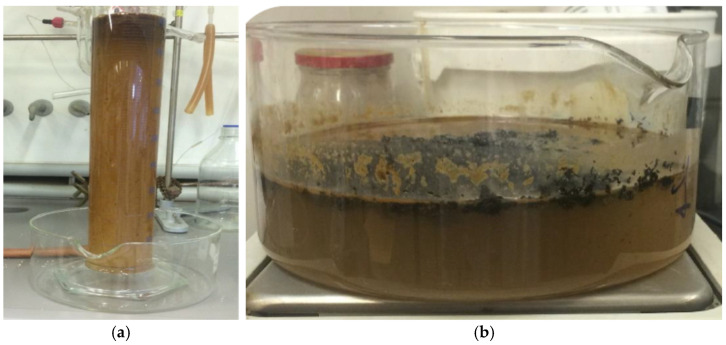
(**a**) Aspect of the real water sample; (**b**) G+ in contact with the real water sample.

Very similarly, a further test was conducted on a real water sample from a groundwater treatment plant located in central Italy. This sample, consisting of two separate phases (Figure 4a), had the following characteristics:

1. A few tens of ppm of Fe, Na, and Ca;
2. High concentrations of hydrocarbons (heavy and light);
3. High concentrations of styrene;
4. A total volume of 930 mL, of which 80 mL of separate phase.

In this case, the absorbent material in loose form (G+ or PP) was put in direct contact with the stirred sample until saturation, as shown in Figure 4b. The quantitative evaluation of the adsorption was carried out by weighing both the material, before and after the contact, and the quantity of residual sample in the crystallizer.

### 2.3. Tank Tests

**Figure 5:**
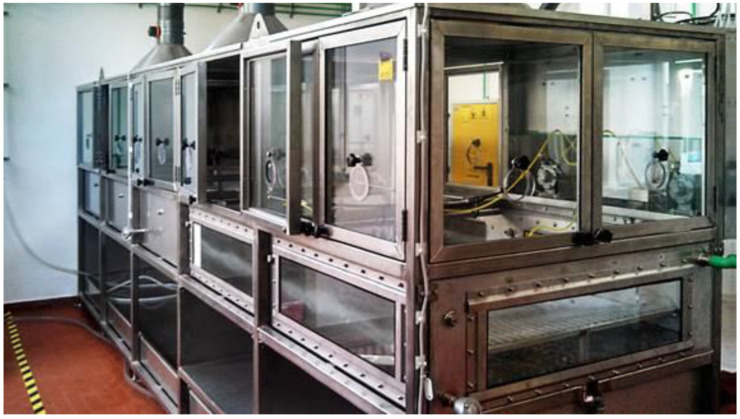
Pilot tank device for oil-adsorbent capacity testing of Grafysorber^®^ G+ and PP booms in dynamic conditions.

To deepen the investigation and confirm the evidence of laboratory tests, some tests were carried out on a larger scale, inside a pilot tank, as shown in Figure 5.

The supporting structure is in AISI 316 steel, the overall structure is 1.84 m high (excluding the suction system), 5 m long, 1.17 m wide, and 35.5 cm deep. At both ends, there are two lateral panels (40 cm wide and 1.032 m long), the first to have the instrumentation inside the aspirated environment (pumps and scale), the second to separate the inductor motor from the tank. A 3-point suction intake system, filtered with activated carbon before leaving, allows the system to be isolated from the laboratory environment. The device is equipped with glass side windows (5 in total) that allow the operator to define and control the level of liquids in the tank and to monitor the device. At one end, controlled by the gear motor, is placed the wave-generating device.

The tests were performed using two-wave intensities of 0.125 and 0.5 Hz, equal to 10 and 50% of the device’s capacity, respectively. The intensity of 10% represents well the situation of a river course while the application of 50% of the wave motion can be more representative of the case of a spill in the open sea. The two conditions are substantially different from the point of view of the contact between the adsorbent material and the liquid phase: In the first case the adsorbent specimen floats on the water and only the lower part is in direct contact with the oil, while in the second case, the waves submerge the specimen, thus increasing the contact between the adsorbent surface and the liquid phase.

Tank tests were carried out with the same diesel used in laboratory tests, while crude oil and kerosene were not used during the tests for safety reasons (CLASS A—Highly flammable liquid) and, as for the crude oil, because the cleaning of the tank would have been extremely difficult.

For both types of induced waves, the tests were carried out with the same logic of laboratory tests, resulting in this case in 5 and 1 mm (iridescence) of diesel above 10 cm of water. To perform the tests, samples were packed as reported in Table 2. Of particular interest was the possibility of comparing the adsorption effectiveness of the booms with the two different fillings (Grafysorber^®^ G+ and PP) in the dynamic conditions described.

Unfortunately, it was not possible to carry out containment tests in the tank, mainly for practical and safety reasons (it would have been necessary to make a boom containing a quantity of G+ that would have been too heavy at saturation and therefore difficult to handle safely).

### 2.4. Containment Tests

**Figure 6:**
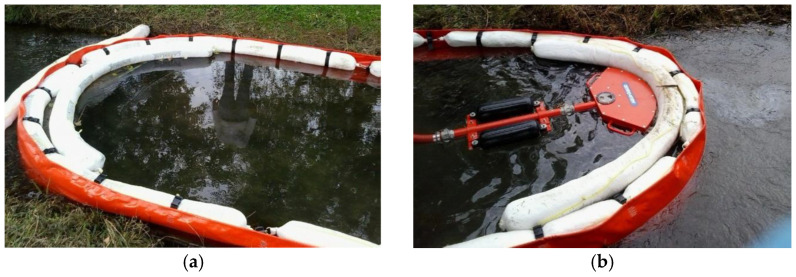
Example of a polypropylene boom (**a**) and oil recovery skimmer (**b**).

To date, many of the interventions in confined waters as rivers involve the use of booms filled with polypropylene to contain and concentrate the oil, which is then sucked through a skimmer. Figure 6 shows an example of this.

In this perspective, it was decided to evaluate the behavior of the Grafysorber^®^ G+ filled booms and compare them with the performances of traditional PP booms. Once filled, the PP boom used in the experiment contained 148 g of PP flake, while the one in Grafysorber^®^ G+ contained 27 g of G+.

**Figure 7:**
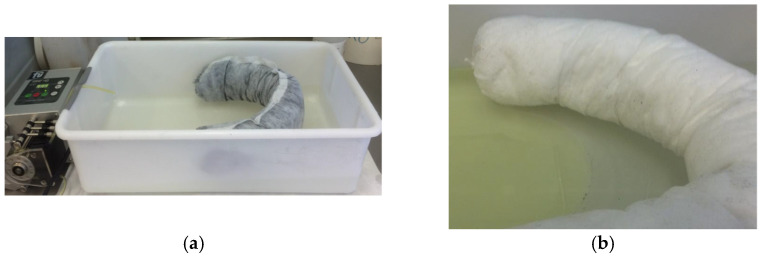
Experimental device for containment tests: (**a**) with a Grafysorber^®^ G+ boom; (**b**) with a PP boom.

To perform the test, a 45 × 24 cm tray containing 3 L of water was used; the boom (length 50 cm, diameter 7.5 cm) was positioned to form a U. Using a peristaltic pump, diesel fuel was introduced onto the surface, with a flow rate of 10 mL/min. The tank was slightly tilted to facilitate the flow of diesel fuel towards the boom (Figure 7). The duration of the test was programmed to know the quantity of diesel fuel introduced into the tank.

## 3. Results

### 3.1. Experimental Tests with Diesel

In the case of water only, the floating material adsorbed an extremely limited quantity (equal to 1/5 of its weight in the case of G+) and no leakage was observed, neither by simple dripping nor by squeezing. On the other hand, by immersing the sample in water up to saturation, the absorbed quantity increased, up to just above the weight of the absorbent, both for G+ and PP.

For the test n.2 (with 1 cm of oil), several adsorption/squeezing cycles were performed (the obtained values are reported in Table 3). The specimen was weighed immediately after adsorption (adsorbed diesel weight + pillow), then the releasable diesel fuel was evaluated after the sample spent 30 min on the mesh grid (weight of released diesel oil); finally, the sample was weighed again after squeezing (weight of the squeezed pillow). The contact with the oil was maintained for 2 min, but saturation was observed already after 6–8 s in the case of G+.

At the first adsorption cycle, a capacity of over 76 g/g was recorded for G+, compared to 13 g/g for PP. The adsorption values in subsequent cycles decreased to around 30 and then c.a. 20 g/g for G+, while they immediately stabilized around 6 g/g in the case of PP.

**Figure 8:**
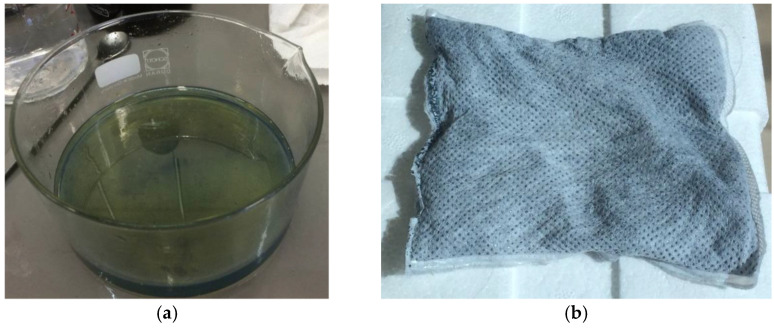
(**a**) Water appearance after test n.2 with Grafysorber^®^ G+ and diesel; (**b**) Pillow containing Grafysorber^®^ G+ after 6 cycles of adsorption/squeezing.

This suggests that, after squeezing, the adsorption capacity of PP is restored to 50% compared to the virgin material. Regarding G+, an approximately constant quantity (about 15%) of diesel remains inside the material after each squeezing—the weight of the cushion after squeezing is constant in all tests, supporting the reproducibility of the same wringing. However, as evident from the values shown in the last column of Table 3, there is a decrease in the absorbent capacities of Grafysorber^®^ G+ after each cycle, probably because the wringing induces changes in the morphology of the material. Indeed, a loss of material from the pillow was observed, as clear in Figure 8a, likely due to the vigorous manual squeezing. After 6 cycles, the G+ cushion appears as in Figure 8b.

**Figure 9:**
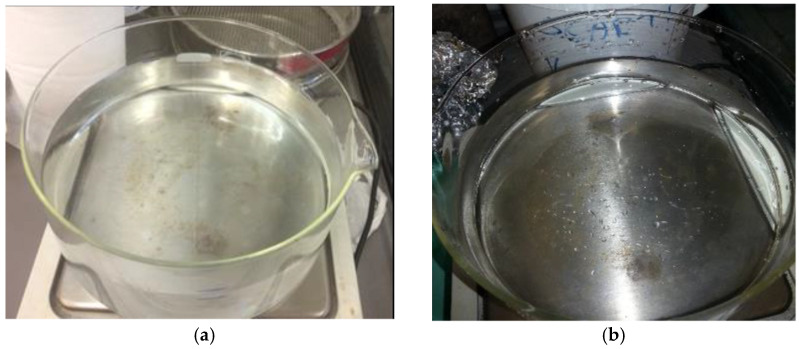
Water appearance after test n.3 with a thin film of diesel using (**a**) Grafysorber^®^ G+; (**b**) PP.

As for test n.3, it used a very thin film of diesel (iridescence) placed on the water using a dry specimen. In this case, the following observations were made for both the sorbent materials:

- kinetics comparable to those of the test n.2 (in the presence of 1 cm of diesel);
- the whole film of diesel fuel is easily absorbed by Grafysorber^®^ G+ (Figure 9);
- no release of diesel was observed when removing the sample from the water.

Therefore, the substantial difference between the two materials lies in the ability to retain diesel, with a value of over 76 g/g for G+ at the 1st cycle compared to about 13 g/g for PP.

### 3.2. Experimental Tests with Crude Oil

As done in the tests conducted with diesel, the G+ performance evaluation was carried out with respect to crude oil, in comparison with the use of PP flakes. Tests were carried out with 1 cm of crude oil on 5 cm of water, and with a thin layer of crude oil. The crude oil used has an API value of 34.1 and a density of 0.8543 g/mL (Table 1).

**Figure 10:**
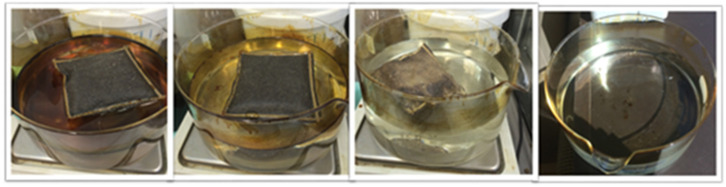
Adsorption sequence related to test n.2 (with 1 cm of oil) using Grafysorber^®^ G+ with crude oil. Evolution over time from left to right.

The results of the test for G+ are shown in Table 4 and Figure 10. The adsorption of crude oil by virgin material was equal to about 74 g/g, but the value decreased to about 30 g/g after the first cycle and subsequently showed a constant decrease down to a value of 24 g/g after the fourth cycle. Considering that the quantity of crude oil remaining inside the pillow after squeezing is always the same, this suggests once again a decrease in the performance of the material, probably due to structural modifications given by the action of the squeezing on the particles themselves. The performance of the material with this crude oil was completely comparable to that shown with diesel fuel and the viscosity values of the two hydrocarbons are comparable to each other.

The test with PP (also reported in Table 4), instead, highlighted the following aspects:

- The PP in contact with the crude oil does not reach saturation unless it is mixed to facilitate the contact of the crude oil with the entire mass of the flake; in other words, no diffusion of crude oil due to capillarity occurs inside the PP, while this was observed for Grafysorber^®^ G+. This could lead to excessive use of PP in the case of rather still waters;
- The adsorption capacity is halved after the first cycle since the wringing restores only 50% of the PP removal capacity.

Placing the PP flake specimen in contact with an iridescent layer of crude oil (about 20 g), it was observed that the material is not able to completely clean the water, leaving a certain amount of crude oil on the surface. In the case of Grafysorber^®^ G+, instead, the crude oil layer was completely removed, leaving the water surface as clear as in Figure 9a.

### 3.3. Experimental Tests with Kerosene

Once again, two tests were performed: one with 1 cm of kerosene on 5 cm of water (equal to about 250 g) and the other with a thin layer of kerosene (20 g). The results of the first test are reported in Table 5. The adsorption of kerosene by the virgin material was equal to about 50 g/g, a value that progressively decreased in the subsequent adsorption/squeezing cycles, down to 19 g/g at the fourth cycle. After wringing, about 10% of kerosene remained inside the material and the dripping loss was minimal.

As regards the comparison tests with PP (also in Table 5), the adsorption of kerosene by the virgin material was equal to about 8 g/g, but the value decreased to about 4 g/g in the subsequent adsorption/wringing cycles. The performances were therefore clearly inferior to those of Grafysorber^®^ G+.

As for the tests with a thin layer of kerosene, by placing the pillow of G+ in contact with the iridescence (about 20 g), it was observed that the material is able to completely clean the water by adsorbing a small amount of water (about 1 g, compared to 0.77 g of Grafysorber^®^ G+ present initially in the pillow). However, in this case, the PP in contact with a layer of kerosene showed similar performances, completely cleaning the water and adsorbing only a small amount of water.

### 3.4. Cumulative Adsorption and Considerations on Laboratory Tests

**Figure 11:**
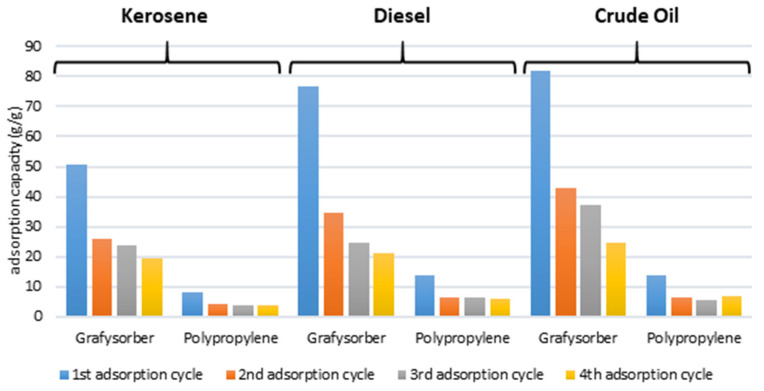
Adsorption capacity after a few regeneration cycles.

G+ showed superior performances with all the tested oils compared to polypropylene in flakes, which also had slower adsorption kinetics (in the order of minutes, compared to seconds for G+). In particular, Grafysorber^®^ G+ pillows showed a maximum adsorption performance with diesel (76.8 g/g) and a minimum with kerosene (50.8 g/g), whereas PP pillows adsorbed a maximum of 13.9 g/g of crude oil and only 8.05 g/g of kerosene (Figure 11). This means that considering a single pillow, the oil adsorption capacity of Grafysorber^®^ G+ is at least 5 times higher than that of PP (in any case at least 80% more, in terms of mass percentage).

Considering repeated cycles, the adsorption values for Grafysorber^®^ G+ were always higher than 19 g/g while the lowest adsorption values for PP were around 4 g/g.

**Figure 12:**
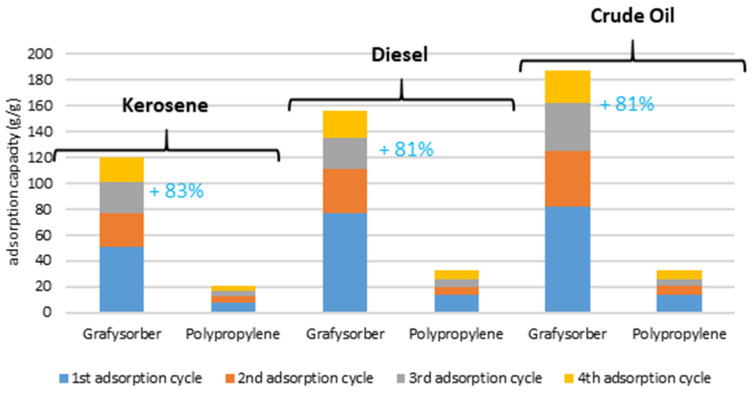
Cumulative adsorption capacity, after 3 regeneration cycles (4 uses).

Considering the cumulative amount of oil adsorbed during the tests (Figure 12), a Grafysorber^®^ G+ pillow used 4 times can adsorb an amount of kerosene, diesel oil, or crude oil of about 120, 156, and 187 times its weight, respectively. Under the same conditions, a polypropylene pillow adsorbs 20, 32, and 33 times the weight of the same oils, respectively.

In percentage, it is confirmed that Grafysorber^®^ G+ has a higher adsorption capacity than PP (81% higher than PP after 3 regeneration cycles) and although the oil adsorption capacity is influenced by the viscosity of the oil, the G+ pillows have proven to be highly performing against low and high viscosity oils. Moreover, in the case of the presence of traces of oil, e.g., as a surface iridescence, polypropylene is not able to satisfactorily absorb the oil unless a slight movement inside the system is created, a limitation not observed in the case of G+.

### 3.5. Tank Test

For these tests, the absorbed amount of oil was calculated taking into account the different quantities of Grafysorber^®^ G+ and PP inside the devices.

As regards the tests with the cushions, there were no differences in the adsorption times for Grafysorber^®^ G+, at 10% or 50% of wave motion, in the presence of a layer of diesel oil of at least 5 mm: adsorption was fast (a few minutes) and equal to 41 g/g in both cases. It is not clear why a reduction of almost 50% was achieved compared to laboratory tests. If the diesel is present as a thin layer, the behavior in the presence of weak and strong waves changes, with the adsorption times becoming longer in the case of weak waves (up to 2 h to have saturation).

Even in the case of tests carried out with the booms, the presence of a diesel layer having a thickness of 5 mm ensured the absence of differences in absorption times at 10% or 50% of wave motion (adsorption remains fast, taking only a few minutes). At the end of the test, the material was not completely saturated, the water was clear, and the adsorption capacity was estimated at 32 g/g. On the other hand, PP showed a slower kinetic, requiring about 20 min to reach an adsorption value of 7 g/g, which is in any case significantly lower than the value estimated for Grafysorber^®^ G+.

### 3.6. Containment Tests

Also in these tests, the behavior of the two materials was different. In the case of PP, over time a widening of the surface patch of the diesel fuel was observed, which always remained detached from the walls of the boom. The latter remained dry throughout the test, which lasted 15 min. At the end of the test, some waves were manually produced leading to contact between the diesel fuel and the PP, with consequent adsorption inside the latter. With a simple calculation, it was estimated that the quantity of adsorbed diesel was 126.2 g, while 126.7 g were placed in the tank.

In the case of Grafysorber^®^ G+, on the other hand, a widening of the patch was not observed, since the diesel fuel introduced into the tank was gradually adsorbed by the graphite-based adsorbent material. In this case, the presence of waves only caused the adsorption of some of the water by Grafysorber^®^ G+, since the diesel was already completely adsorbed during the 20 min of testing. The presence of water was deduced by the fact that the boom weighed 50 g more than the sum of diesel fuel (170 g) and Grafysorber^®^ G+ (27 g) at the end.

It is therefore clear that unlike the systems currently used, in situations of calm water Grafysorber^®^ G+ works not only as a barrier, but also as an oil adsorbent, and it is therefore not necessary to remove the oil accumulated near the barrier with other means.

### 3.7. Experiments with Real Wastewater

The test performed with real water gave an adsorption value of the supernatant phase equal to 15 g of contaminant absorbed per g of Grafysorber^®^ G+, against a null value for PP flakes.

As described in the materials and methods section, for the quantitative evaluation, in addition to the residual content of the water sample in the crystallizer before and after contact, the material itself was weighed. This was possible because, once saturated, the Grafysorber^®^ G+ appears almost like a paste and is therefore easily separable from the aqueous solution.

For scientific completeness, we also tried to use a bearing like the one considered for the oil spill tests. In this case, it has been observed that the external NWF blocks the diffusion inside the pillow, hindering the adsorption of the supernatant by Grafysorber^®^ G+.

### 3.8. Economic Considerations

Table 6 shows the data relating to the cost for the recovery of 1 kg of the various oils using Grafysorber^®^ G+ or PP. The starting data used relate to laboratory tests, as they are the most complete. The cost per kg of PP was obtained from the net [40] and is around 13 €/kg for this type of use, while that of Grafysorber^®^ G+ is around 50 €/kg, although strongly depending on various factors such as the requested quantity, as indicated by Directa Plus.

The percentage savings are around 32% for diesel, 29% for crude oil, and 39% for kerosene.

The same accounts were also made considering the quantity of oil retained over 4 adsorption/wringing cycles by the same specimen. The high affinity for crude oil is particularly evident while for diesel and kerosene there is a decrease in percentage savings with values that go down to 20 and 34% respectively, for crude oil, there is an increase of up to 30%.

Directa Plus S.p.A. is currently looking for technological solutions that allow reduction of cost while guaranteeing products of the same quality.

## 4. Conclusions

In this work, the performance of Grafysorber^®^ G+, a graphite-based material (Directa Plus S.p.A., Lomazzo (CO), Italy) was evaluated with respect to polypropylene flakes, through a series of laboratory and tank tests, using oils with different chemical and physical properties (diesel, crude oil, and kerosene) to remove oil in the event of an oil spill and water treatment. In addition to the ability to retain oil, the possibility of reusing the material after regeneration via squeezing was also evaluated.

Laboratory tests attested the oil adsorption capacity of G+ to be at least 5 times higher than that of PP, an advantage that increases considering the possibility of wringing and reusing the material, with G+ adsorbing from 120 to 187 times its weight (depending on the type of oil) in a cycle of 4 uses, against 20–33 times for PP.

Similar results were also obtained in (dynamic) tank tests, where adsorption with PP was generally slower and less effective than G+.

Regarding the confinement tests, results showed that Grafysorber^®^ G+ works not only as a barrier but also absorbing the oil, thus avoiding the need to remove the latter using other means when accumulated.

The possibility of using the Grafysorber^®^ G+, directly in its loose form, was also evaluated: using a real water sample, G+ captured the supernatant phase in a quantity equal to 15 g/g, whereas PP flakes showed zero adsorption in the same conditions.

In conclusion, for all the oils tested, the performance of G+ was significantly higher than that of polypropylene flakes, showing better hydrophobic properties, greater adsorbent capacity, and faster adsorption kinetics (in the order of seconds, compared to minutes for PP). In the case of the presence of traces of oil, or as a surface iridescence, PP was not able to satisfactorily absorb the oil unless a slight movement inside the system is created.

As for regeneration, cyclical use tests have confirmed certain reusability of G+, albeit with a significant loss of adsorbing capacity. This highlights the limits of regeneration via squeezing, which do not allow complete regeneration of the material and imply damage to the structure of the material due to mechanical stress. However, other solutions attempting to maximize the G+ regeneration—and thus the adsorption capacity in subsequent uses—must guarantee the preservation of the intrinsic properties of G+ (above all the absence of chemicals and toxic substances) and the possibility of recovering the adsorbed oil, avoiding at the same time to undermine the environmental and economic sustainability of the treatment. This issue is far from trivial and will be addressed in an ad hoc experimentation.

In addition, based on the experimental results obtained, some indicative considerations on the cost deriving from the treatment of the various oils considered show a certain advantage in the use of the Grafysorber^®^ G+ also from an economic point of view.

However, it should be emphasized that the performance reported in this study refers to an experimental laboratory campaign, therefore in ideal conditions. Under real conditions, even if the effectiveness of the G+ from a technical point of view is still higher [9], as well as the advantage in terms of environmental sustainability, the gap between the two materials tends to reduce, so the real advantage in using the G+ should, in any case, be assessed on a case-by-case basis.

## Figures and Tables

**Table 1:** Properties of the oils used for laboratory tests.

Oil Type	API	Density (g/mL)	Viscosity (cP @ 20 °C)
Crude Oil	34.1	0.8543	7.0
Diesel	---	0.8447	5.8
Kerosene	---	0.8107	1.9

**Table 2:** Samples for tank tests.

Adsorbent	Format	Length (m)	Diameter (cm)	Initial Weight (g)
Grafysorber^®^	Boom	50	7.5	27
Polypropylene	Boom	50	7.5	148
Grafysorber^®^	Pillow	50	---	65

**Table 3:** Diesel adsorption tests with Grafysorber^®^ G+ and Polypropylene.

	Adsorbent	Initial Weight (g)	Weight after Squeezing (g)	Adsorbed Diesel (g)	Adsorbed Diesel/Sorbent (g/g)
1° Cycle	Grafysorber^®^	0.78	6.45	59.9	76.8
	Polypropylene	10.0	31.8	136	13.6
2° Cycle	Grafysorber^®^	6.45	6.00	27.0	34.6
	Polypropylene	31.8	33.0	63.2	6.32
3° Cycle	Grafysorber^®^	6.00	4.93	19.3	24.7
	Polypropylene	33.0	30.3	62.0	6.20
4° Cycle	Grafysorber^®^	4.93	5.19	16.4	20.1
	Polypropylene	30.3	31.5	61.1	6.11

**Table 4:** Crude oil adsorption tests with Grafysorber^®^ G+ and Polypropylene.

	Adsorbent	Initial Weight (g)	Weight after Squeezing (g)	Adsorbed Crude Oil (g)	Adsorbed Crude Oil/Sorbent (g/g)
1° Cycle	Grafysorber^®^	0.80	7.68	59.6	74.5
	Polypropylene	10.0	33.2	139	13.9
2° Cycle	Grafysorber^®^	7.68	9.18	34.5	43.1
	Polypropylene	33.2	31.7	62.4	6.24
3° Cycle	Grafysorber^®^	9.18	8.37	30.0	37.5
	Polypropylene	31.7	32.0	56.2	5.62
4° Cycle	Grafysorber^®^	8.37	9.04	19.9	24.9
	Polypropylene	32.0	31.8	68.3	6.83

**Table 5:** Kerosene adsorption tests with Grafysorber^®^ G+ and Polypropylene.

	Adsorbent	Initial Weight (g)	Weight after Squeezing (g)	Adsorbed Kerosene (g)	Adsorbed Kerosene/Sorbent (g/g)
1° Cycle	Grafysorber^®^	0.77	7.80	39.1	50.8
	Polypropylene	10.0	32.4	80.5	8.05
2° Cycle	Grafysorber^®^	7.80	8.38	20.1	26.1
	Polypropylene	32.4	30.4	43.2	4.32
3° Cycle	Grafysorber^®^	8.38	8.13	18.2	23.6
	Polypropylene	14.07	28.2	39.0	3.90
4° Cycle	Grafysorber^®^	8.13	7.22	14.9	19.3
	Polypropylene	28.2	29.7	39.3	3.93

**Table 6:** Indicative costs for the recovery of 1 kg of the various oils considered by using Grafysorber^®^ G+ and Polypropylene.

	Adsorbent	1° Cycle		Total (4 Cycles)	
		Grafysorber^®^	Polypropylene	Grafysorber^®^	Polypropylene
Diesel	Adsorbing Capacity (g/g)	76.8	13.6	156.2	32.23
	Specific Cost (€/kg)	0.65	0.96	0.32	0.40
	Savings	32.3%	---	20.0%	---
Crude Oil	Adsorbing Capacity (g/g)	74.5	13.9	180.0	32.59
	Specific Cost (€/kg)	0.67	0.94	0.28	0.40
	Savings	28.7%	---	30.0%	---
Kerosene	Adsorbing Capacity (g/g)	50.8	8.05	119.8	20.2
	Specific Cost (€/kg)	0.98	1.61	0.42	0.64
	Savings	39.1%	---	34.4%	---

## Data Availability

Data is contained within the present article.
